# Chemical and Molecular Approach to Tumor Metastases

**DOI:** 10.3390/ijms19030843

**Published:** 2018-03-14

**Authors:** Alberta Bergamo, Gianni Sava

**Affiliations:** 1 Callerio Foundation Onlus, 34127 Trieste, Italy; a.bergamo@callerio.org; 2 Department of Life Sciences, University of Trieste, 34127 Trieste, Italy

Tumours are not merely masses of abnormally proliferating cancer cells. Today, we have a clearer view of cancer complexity in which the participation of cancer and host cells leads to a tremendous heterogeneity of neoplastic diseases concerning the genetics, epigenetics, proteomics and biochemistry of the tumour [1]. Such intra-tumour heterogeneity provides the basis for inter-metastatic heterogeneity among different metastatic lesions of the same patient, each originating from a founder cell, or small group of cells, with a very different mutation kit, and likely originating from different and distinct primary tumour areas [1]. This situation has important implications regarding chemotherapeutic sensitivity and responses. In addition, the offspring of the founder cell(s) can generate heterogeneity among the cells of an individual metastasis, affecting the response to systemic therapies and providing the seeds for drug resistance.

Correspondingly, drug treatment is progressively shifting from the use of chemicals producing toxics effects in general processes of cell division, with the goal of killing the tumour cell, to compounds targeting specific cell behaviours with the goal of disarming the malignancy of the tumour cell (Figure 1) [2,3]. This new strategy implies the use of novel systems of drug design, particularly those concerning biological drugs leading to compounds capable of targeting specific molecules expressed only by selected tumour cells [4,5,6]. The main aim of this novel era of drug development is the response to the need of overcoming the heavy toxicity of the “conventional” chemotherapy that poorly distinguishes between cancer and healthy cells, therefore, causing severe side effects that often hamper the compliance of the patient.

Metastasis is the primary target for cancer chemotherapy, independently of the kind of drug being used. The main reason is that the primary tumour can often, if not always, be aggressed by surgery and/or radiotherapy whereas metastases, spread in several tissues and organs, are believed to be better reached with drugs that follow the pharmacological rules of distribution in the body.

The generation of these novel drugs, whether selective and specific monoclonal antibodies or small organic molecules, requires a deep knowledge of the nature of the tumour cell and particularly of tumour metastasis. Similarly, to the primary lesions, tumour metastases are characterised by the interactions with healthy cells and extracellular matrix leading to a complex microenvironment in dynamic evolution but extremely important for the metastatic growth [7,8].

The twin research that joins the biochemistry and molecular biology of cancer metastasis with the study of novel targets and novel approaches to combat their growth is even more mandatory in a scientific era in which the improvements of human health are transforming cancers into chronic diseases and therefore significantly prolonging the life-time expectancy.

## Figures and Tables

**Figure 1:**
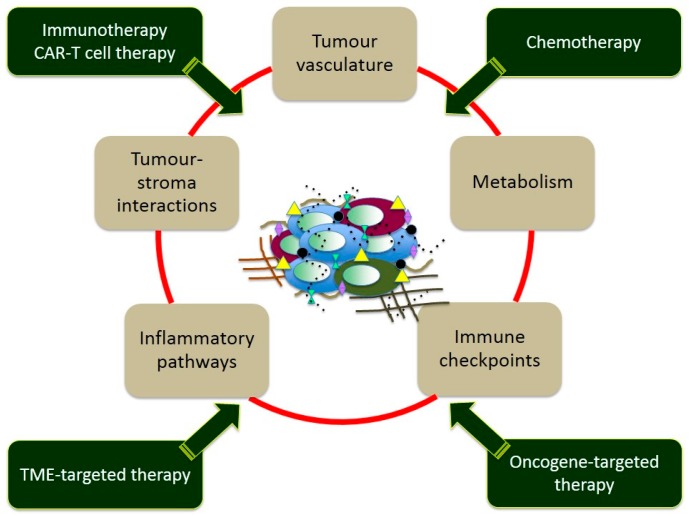
Graphical representation of the tumour/metastasis heterogeneity and of the main targets and therapeutic approaches (CAR-T = Chimeric Antigen Receptor T cell; TME = Tumour Micro Environment).
